# Proanthocyanidins Inhibit Neuroinflammation in High-Fat-Induced Obese Mice by Modulating Intestinal Flora and Their Metabolites

**DOI:** 10.3390/nu18030431

**Published:** 2026-01-28

**Authors:** Min Yao, Xiaotong Pang, Hailiang Wang, Cunxi Nie, Ruolin Huang, Fang Wang, Heng Zhao, Wenna Tang, Yueran Hao, Yixin Ren

**Affiliations:** 1 School of Medcine, Shihezi University, Shihezi 832000, China; ym408587713@sina.com (M.Y.); 18336603826@163.com (R.H.); sss014788888888@163.com (H.Z.); 15839684686@163.com (W.T.); 13655600884@163.com (Y.H.); renyixin001@163.com (Y.R.); 2 Bingtuan Key Laboratory for Efficient Utilization of Non-Grain Feed Resources, College of Animal Science and Technology, Shihezi University, Shihezi 832000, China; pxt1524896983@sina.com (X.P.); hlwangup@163.com (H.W.); 20222013004@stu.shzu.edu.cn (F.W.)

**Keywords:** high-fat diet, neuroinflammation, cognitive performance, gut flora, gut metabolite, proanthocyanidin

## Abstract

**Background/Objectives**: The effect of proanthocyanidins (PAs) on neuroinflammation through the modulation of colonic microflora and their metabolites was investigated in obese mice fed a high-fat diet (HFD). **Methods**: Thirty healthy male C57BL/6J mice of similar body weight were randomly divided into control (CON), high-fat diet (HFD), and proanthocyanidin (PA_HFD) groups. HFD and PA_HFD groups were fed an HFD, whereas the CON group was fed a basic diet for 8 weeks. Subsequently, the CON and HFD groups were administered equal doses of saline, and the PA_HFD group was administered PA (100 mg/kg/day) daily. We evaluated microbial changes through gut microbiota richness and probiotic relative abundance, analyzed metabolite variations via non-targeted metabolomics and pathway enrichment, assessed neuroinflammation via related gene expression, and measured cognitive function using platform crossing frequency and target quadrant time in the Morris water maze, where longer duration and more crossings indicate better cognition. **Results**: Body weight was significantly lower in the PA_HFD group than in the HFD group. In the PA_HFD group, fewer inflammatory and hepatic fat cells were observed, and hepatocellular edema was alleviated. PA significantly decreased total cholesterol, low-density lipoprotein, IL-1β, TNF-α, lipopolysaccharide, and Lc3 expression and increased Sirt1 and FGF21 expression in hippocampal tissue (*p* < 0.01). PA significantly altered the abundance of colonic microbiota (*p* < 0.01), including phyla *Patescibacteria* and *Bacteroidota* and genera *Lactobacillus* and *Akkermansia*. KEGG analysis revealed that differences in metabolite profiles between CON and HFD groups were reflected in glycerophospholipid metabolism, while those between HFD and PA_HFD groups were in steroid hormone biosynthesis and tryptophan metabolism. Metabolomic analysis demonstrated that changes in metabolites and microbiota were significantly correlated with neuroinflammation. **Conclusions**: In conclusion, PAs play a role in modulating neuroinflammation, colonic microflora, and colonic metabolites in mice and have a mitigating effect on cognitive decline in HFD-induced obese mice.

## 1. Introduction

Obesity results from an energy imbalance and is predominantly characterized by the excessive accumulation of body fat [1]. As a serious global health concern, obesity is associated with numerous health complications, including cardiovascular diseases, diabetes, hyperlipidemia, nonalcoholic fatty liver disease, and cognitive dysfunction [2,3,4]. The primary causes of obesity include non-modifiable factors, such as genetics (e.g., gene expression in the leptin–melanocortin pathway) and hypothalamic damage, and modifiable factors, such as epigenetics, physical inactivity, high-calorie intake, obesogenic intrauterine environment, sleep deprivation, medications, medical conditions, socioeconomic status, ethnicity, psychosocial stressors, endocrine-disrupting chemicals, gastrointestinal microbial composition, and postnatal influences [5].

In animal models, high-fat diets (HFDs) have been extensively investigated for their role in inducing obesity and related syndromes, such as metabolic syndrome and insulin resistance [6]. Long-term consumption of HFDs induces insulin resistance and neuroinflammation, which may lead to deficits in long-term memory formation and cognitive impairment [7,8,9]. Moreover, HFDs are associated with altered lipid metabolism and dysbiosis of the gut microflora [10]. Sodium propionate treatment has been shown to enhance lipid metabolism in HFD-fed mice, potentially through the modulation of intestinal flora [11].

The microbiota–gut–brain axis is a bidirectional signaling mechanism between the gastrointestinal tract and the central nervous system [12], which facilitates communication between the gut microbiota and brain [13]. Through this pathway, metabolic disorders such as obesity can lead to brain damage [14]. Some gut microbes, such as *Clostridium butyricum*, have been reported to act through this pathway to exert neuroprotective effects against obesity-related cognitive impairment and neurodegenerative diseases [15].

Proanthocyanidins (PAs), a class of flavonoid compounds, are oligomers or polymers of flavan-3-ols derived from several plants and can be classified into type A and B PAs [16]. PAs possess various biological functions, including antioxidant [17], anti-inflammatory [18], antimicrobial [19], antitumor [20], anti-obesity [21], anti-hyperglycemia [22], and lipid-lowering [23] properties. HFDs can promote the expression of inflammatory factors by activating the MAPK and NF-κB signaling pathways [24]. Grape seed PAs can prevent mild inflammation by modulating the expression of cytokines C-reactive protein [CRP], interleukin [IL]-6, tumor necrosis factor [TNF-α], and lipocalin) in HFD-fed rats [25]. Furthermore, PAs prevent ethanol-induced cognitive deficits by inhibiting oxidative stress and inflammatory responses in the brains of adults [26].

A growing body of research has established a correlation among gut microbiota dysbiosis, cognitive dysfunction, and inflammatory responses [27]. PA supplementation prevents weight gain and alters gut microbiota composition in HFD-induced obese mice [28]. Therefore, the objective of this study was to investigate whether PAs could alleviate HFD-induced cognitive decline in mice by inhibiting neuroinflammation and modulating colonic microflora and their metabolites.

## 2. Materials and Methods

### 2.1. Animals and Experimental Design

Thirty healthy male C57BL/6J mice (3 weeks old) with similar body weights (12–13 g) were purchased from Henan Skibbes Biotechnology Co., Ltd. (Huaxian, China) Mice were randomly divided into control (CON), high-fat diet (HFD), and supplementation with proanthocyanidins in a high-fat diet (PA_HFD) groups, with 10 mice in each group.

For the first 8 weeks of the trial, the HFD and PA_HFD groups were fed a purified HFD (Huafukang Bio-Technology Co.), (Beijing, China) with a fat-to-energy ratio of 60%, and the CON group was fed a basic diet (Huafukang Bio-Technology Co.) with a 10% fat-to-energy ratio. From week 9 to week 15, mice in the CON and HFD groups were orally administered 200 μL of saline daily per mouse, while mice in the PA_HFD group received 200 μL of PA (100 mg/kg/day) daily, The dosage of PA supplementation was determined with reference to several studies, which indicate that a PA dose of 100 mg/kg/day significantly alleviates damage induced by high-fat diet and oxidative stress in rats and mice [29,30,31]. PAs were purchased from Beijing Solepol Technology Co., Ltd. (Catalog P7230, Lot 601l032, CAS: 4852-22-6), (Beijing, China). The PAs were derived from grape seeds, which are of type B, with a purity of 95%; the content per bottle is 5 g.

At the end of week 15, cognitive function was monitored using the water maze test. The diameter and height of the water maze pool were 100 cm and 40 cm, respectively. The platform had a diameter of 10 cm. For the first 5 days, the mice were trained for cognitive function analysis. On the 6th day, the platform was removed, and the stay time in the target area and the number of times the platform was crossed were tested to evaluate cognitive function. Longer time spent in the target area and a higher number of crossings by mice indicate better cognitive function.

The handling of all experimental animals was approved by the Bioethics Committee of Shihezi University (No. A2023-320), and all experiments were conducted in accordance with the relevant guidelines and regulations of the ARRIVE guidelines (https://arriveguidelines.org).

Study Design and Bias Control: Investigators involved in outcome assessment (histological scoring, biochemical assays) and data analysis were blinded to the group allocation (HFD, control or PA_HFD) throughout the experiment. Group identities were revealed only after all analyses were completed.

### 2.2. Mouse Behavioral Testing

At the end of the experimental intervention, all mice underwent the Morris water maze (MWM), which is used to assess their learning, memory, and other cognitive functions.

The Morris Water Maze is a classic test for evaluating spatial learning and memory abilities in mice. It consists of a place navigation test (5 days) and a spatial probe test (1 day). The main body of the water maze is an opaque white circular pool (d = 100 cm, h = 40 cm). The Tracking Master V5 ethological monitoring system divides the pool into four quadrants, and a hidden platform (d = 10 cm) is placed 1 cm below the water surface in one quadrant as the escape target for the mice, which is recorded as the target quadrant. During the first four days of the water maze test, i.e., the place navigation test, each mouse is placed into the water facing the pool wall from each of the four quadrants sequentially to locate the submerged hidden platform. If a mouse fails to find the platform within 60 s, it is manually guided to the platform and allowed to remain there for 15–30 s. The time required for each mouse to reach the platform is recorded as the escape latency using a video monitoring system. A shorter latency indicates better acquisition of learning and memory in the water maze. On the fifth day, the escape platform is removed, and the mice undergo a 1 min free exploration session in the pool without the platform, known as the spatial probe test. The time spent in the target quadrant, the distance traveled, and the number of platform crossings are recorded. After each mouse completes the test, its fur is gently dried with a towel to reduce stress. Longer time spent in the target quadrant and a higher number of platform crossings indicate better retention of spatial memory regarding the platform’s location.

### 2.3. Sample Collection

Food intake and body weight were recorded weekly. At the 16th week, all mice were fasted for 12 h, followed by intraperitoneal injection of chloral hydrate anesthetic. Blood was collected from the tail, and serum was prepared via centrifugation at 1006× *g* for 10 min at 4 °C. The liver, abdominal fat, and colon tissues were fixed with 4% paraformaldehyde. Part of the liver, hippocampus, abdominal fat, colon tissues, and colon contents were rapidly frozen in liquid nitrogen and stored at −80 °C until needed for the analysis of inflammatory factors, fluorescence quantification, microbiota and metabolomics.

### 2.4. Morphologic Analysis of Abdominal Fat, Colon, and Liver

Abdominal fat, colon, and liver were fixed in 4% paraformaldehyde, dehydrated in a series with ethanol, made transparent with xylene, and embedded in paraffin. The embedded wax blocks were sectioned and stained with hematoxylin and eosin (H&E). The morphology of the liver, colon, and abdominal fat tissues was observed under a microscope (OlymbusBX53, Tokyo, Japan).

### 2.5. Measurement of Serum Biochemical Indices

Serum biochemical indices were analyzed at the First Affiliated Hospital of Shihezi University using an automatic biochemical analyzer (Roche Cobas-c702, Shanghai, China). The indicators measured included alanine aminotransferase (ALT), aspartate aminotransferase (AST), postprandial blood glucose, triglyceride (TG), total cholesterol (TC), low-density lipoprotein (LDL), and high-density lipoprotein (HDL).

### 2.6. Hippocampal Tissue Inflammatory Factor Assay

The levels of IL-1β, lipopolysaccharide (LPS), and TNF-α in the hippocampal tissue were assayed with an ELISA kit following the operating instructions. The kits were purchased from (Jiangsu Jingmei Biotechnology Co., Yancheng, China).

### 2.7. qRT-PCR Analysis of Gene Expression in Hippocampal Tissue

Total RNA was extracted from hippocampal tissues using the TransZol Up Plus RNA Kit (Beijing All Style Gold Biotechnology Co., Ltd., Beijing, China), and the purity and integrity of the RNA were assessed using a Thermo Fisher Nanodrop2000 visible spectrophotometer (Thermo Fisher Scientific Inc., Waltham, MA, USA). RNA was reverse transcribed into cDNA using the HiFiScript cDNA Synthesis Kit (Beijing Kangwei Century Biotechnology Co., Ltd., Beijing, China). Primers were designed using Primer 5 (Table 1) and synthesized by Xinjiang Youkang Biotechnology Co., Ltd. (Urumqi, China). *ACTB* was used as an internal reference gene, and the PerfectStart Green qPCR SuperMix kit (Beijing AllStyle Gold Biotech Co., Ltd., Beijing, China) and LightCycler 96 system (Roche Applied Science, Penzberg, Germany) were used for the qRT-PCR. Relative quantification of the above genes was performed using the 2^−ΔΔCt^ assay. PCR amplification conditions included pre-denaturation at 94 °C for 30 s, denaturation at 94 °C for 45 times for 5 s, annealing at 60 °C for 15 s, and extension at 72 °C for 10 s.

### 2.8. Colonic 16S rRNA High-Throughput Sequencing

Total microbial genomic DNA from colon contents was extracted using an E.Z.N.A.^®^ soil DNA kit (Omega Bio-tek, Norcross, GA, USA), and the quality of the extracted genomic DNA was tested using 1% agarose gel electrophoresis. Then PCR amplification was carried out, and the PCR-recovered products were detected and quantified using the Quantus Fluorometer (Promega, Madison, WI, USA). The purified PCR products were used to construct libraries using the NEXTFLEX Rapid DNA-Seq Kit (Revvity Signals, Austin, TX, USA), and sequenced using the NovaSeq PE250 platform of Illumina Inc. (Shanghai Meiji Biomedical Technology Co., Ltd., Shanghai, China).

### 2.9. Colonic Untargeted Metabolomics Analysis

A 50 mg sample of colon contents was weighed, and 400 μL of extraction solution (methanol/water = 4:1, containing 0.02 mg/mL of the internal standard L-2-chlorophenylalanine) was added for metabolite extraction, which was carried out by grinding for 6 min using a low-temperature (−10 °C, 50 Hz) cryo-tissue grinder followed by low-temperature ultrasonic extraction for 30 min (5 °C, 40 kHz). The samples were then allowed to stand at −20 °C for 30 min. Finally, the samples were centrifuged at 13,000× *g* for 15 min at 4 °C, and the supernatant was removed to an injection vial with an internal cannula for analysis. Liquid Chromatography-Mass Spectrometry was performed at Shanghai Majorbio Biomedical Technology Co., Ltd. (Shanghai, China), and the specific assay procedure has been reported previously [32].

### 2.10. Statistical Analysis

Data were analyzed using SPSS software (version 22.0), and the results are expressed as mean ± standard error. *p* < 0.05 indicated significant differences, and *p* < 0.01 indicated highly significant differences. Significance was determined using one-way ANOVA with Duncan’s test and graphically plotted using Origin 2021.

## 3. Results

### 3.1. Effects of PAs on Body Weight and Cognitive Function of Mice

**Figure 1:**
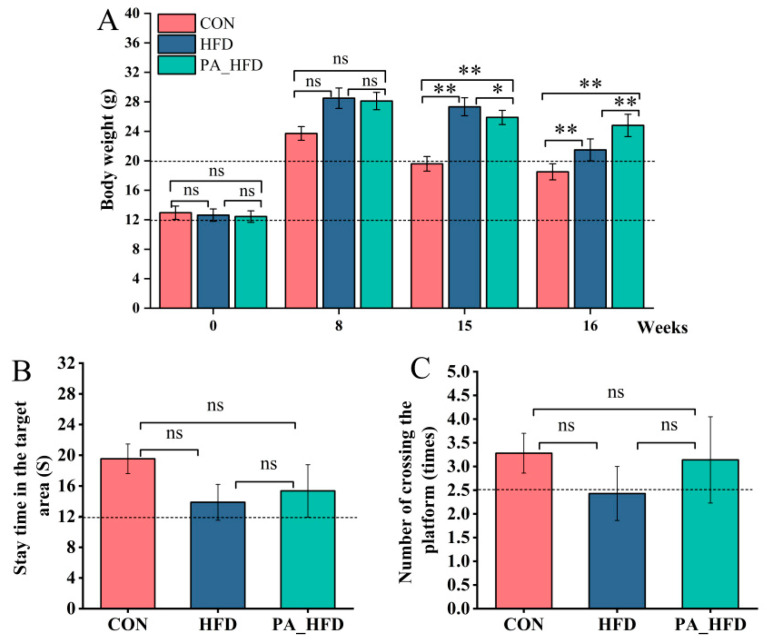
Effects of PAs on body weight and cognitive function in mice. (**A**) Body weight; (**B**) Time that mice stayed in the target area; (**C**) Number of times a mouse crossed the platform within 60 s. *n* = 10, * *p* < 0.05, ** *p* < 0.01. ns indicates insignificant difference. PA, proanthocyanidin.

At the 8th week of the experiment, the mice in both the HFD and PA_HFD groups were heavier than those in the CON group, indicating successful establishment of a high-fat mouse model. However, there was no significant difference in body weight between all groups (*p* > 0.05) (Figure 1A). At the 16th week, the body weight of mice in the PA_HFD group was significantly lower than that in the HFD group (*p* < 0.05). Compared to those in the HFD group, the time spent in the target area and the number of platform crossings were both increased in the PA_HFD group. However, no significant changes were observed. (*p* > 0.05) (Figure 1B,C).

### 3.2. Effects of PAs on the Morphology of Liver, Abdominal Fat, and Colon in Mice

**Figure 2:**
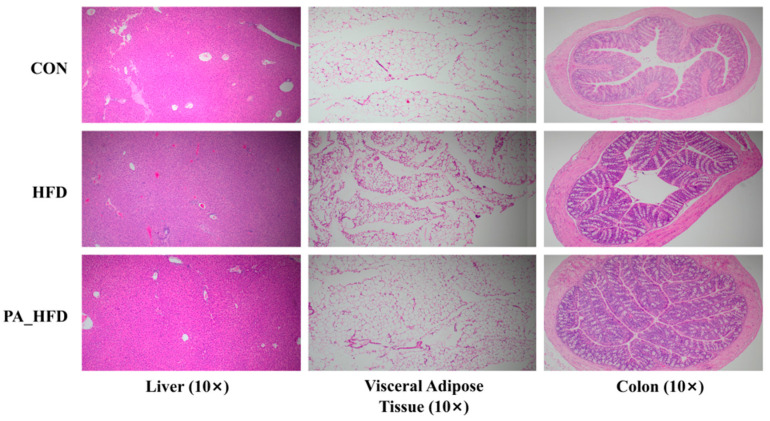
Effects of PAs on the morphology of the liver, abdominal fat, and colon in mice assessed through H&E staining (original magnification: 10×). *n* = 6, H&E, hematoxylin and eosin.

The livers of the mice in the CON group were essentially free of inflammation, whereas those in the HFD group showed increased adipocytosis, steatosis, and hepatocyte edema (Figure 2). The PA_HFD group exhibited reduced hepatic fat cells and attenuated hepatocellular edema compared to the HFD group. There was almost no vacuolar fusion in the abdominal fat of mice in the CON group, whereas the HFD group showed a large number of fractured and fused vacuoles and inflammatory cell infiltration. In addition, compared to the HFD group, the PS group exhibited only partially fused vacuoles and a few inflammatory cells. The CON and PA_HFD groups had more cup cells and fewer inflammatory cells in the colon, whereas the HFD group had more vacuoles and fewer cup cells in the intestinal mucosa.

### 3.3. Effect of PAs on Serum Biochemistry in Mice

**Figure 3:**
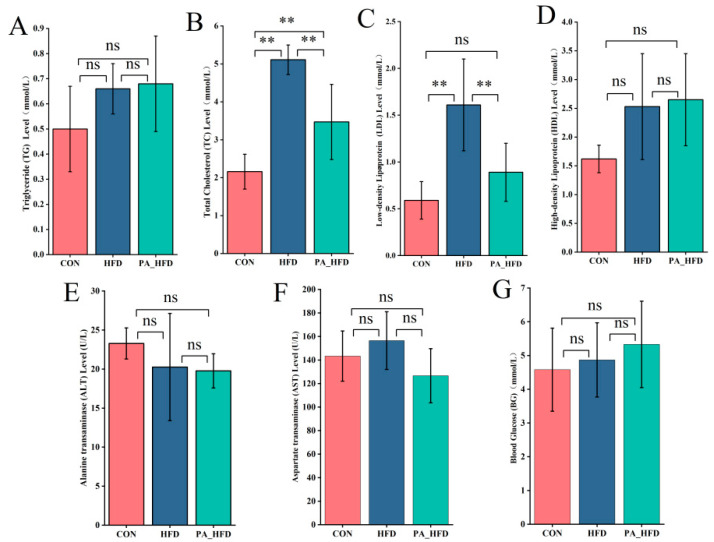
Effects of PAs on serum biochemistry in mice. (**A**) Triglycerides (TG); (**B**) Total cholesterol (TC); (**C**) Low-density lipoprotein (LDL); (**D**) High-density lipoprotein (HDL); (**E**) Alanine aminotransferase (ALT); (**F**) Aspartate aminotransferase (AST); (**G**) Blood glucose (BG). *n* = 6, ** *p* < 0.01. ns indicates insignificant difference.

The serum levels of TC and LDL were significantly lower in the PA_HFD group than in the HFD group (*p* < 0.01) (Figure 3). PAs decreased the enzymatic activities of ALT and AST in the serum; however, the differences were not significant (*p* > 0.05). In addition, the TG and blood glucose levels were higher in the HFD and PA_HFD groups than in the CON group (*p* > 0.05).

### 3.4. Effects of PAs on Inflammatory Factors in Hippocampal Tissue

**Figure 4:**
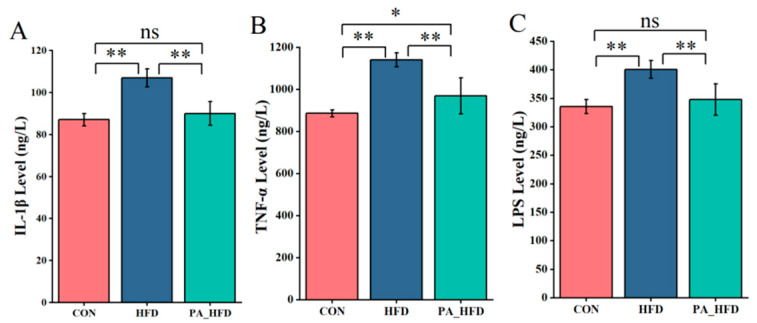
Effects of PAs on inflammatory factors in mouse hippocampal tissue. (**A**) Interleukin 1β (IL-1β); (**B**) Tumor necrosis factor (TNF-α); (**C**) Lipopolysaccharide (LPS). *n* = 6, * *p* < 0.05, ** *p* < 0.01. ns indicates insignificant difference.

The levels of IL-1β, TNF-α, and LPS in hippocampal tissue were significantly lower in the PA_HFD group than in the HFD group (*p* < 0.01) (Figure 4). However, compared with those in the CON group, the levels of IL-1β and LPS did not significantly change (*p* > 0.05) and the TNF-α level increased (*p* < 0.05) in the hippocampal tissue of the PA_HFD group (Figure 4).

### 3.5. Effects of PAs on Gene Expression in Hippocampal Tissue

**Figure 5:**
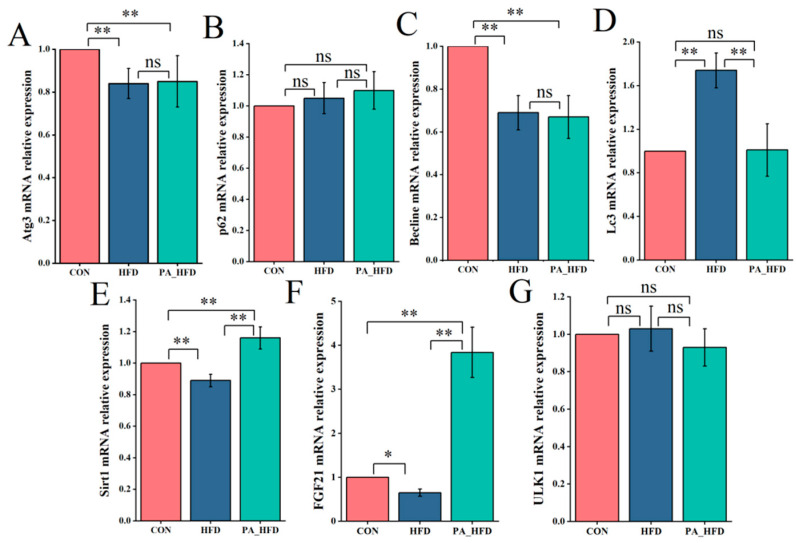
Effects of PAs on gene expression in mouse hippocampal tissue. (**A**) Atg3 expression; (**B**) p62 expression; (**C**) Becline expression; (**D**) Lc3 expression; (**E**) Sirt1 expression; (**F**) FGF21 expression; (**G**) ULK1 expression. *n* = 6, * *p* < 0.05, ** *p* < 0.01. ns indicates insignificant difference.

The expression of Atg3 and Becline was significantly lower in the HFD and PA_HFD groups than in the CON group (*p* < 0.01). However, the expression of Sirt1 and FGF21 was significantly increased in the treatment groups (*p* < 0.01) (Figure 5). In addition, Lc3 expression was significantly decreased (*p* < 0.01), and Sirt1 and FGF21 expression was significantly increased (*p* < 0.01) in the PA_HFD group compared to those in the HFD group.

### 3.6. Effect of PAs on Alpha and Beta Diversity of Mouse Colonic Microbes

**Figure 6:**
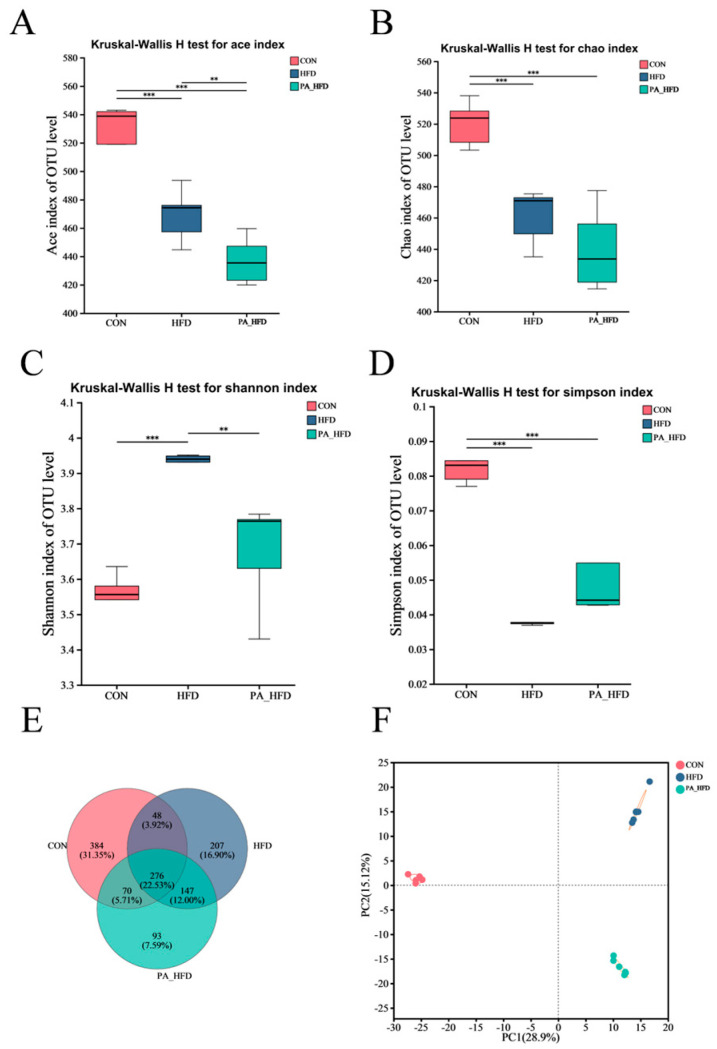
Effects of PAs on microbial α and β diversity in mouse colon. (**A**) Ace richness index; (**B**) Chao richness index; (**C**) Simpson diversity index; (**D**) Shannon diversity index; (**E**) Species Venn plot; (**F**) Principal component analysis (PCA) plot. *n* = 6 ** *p* < 0.01, *** *p* < 0.001.

Following microbiota sequencing analysis of mouse colon contents, we characterized microbial community richness using ACE and Chao indices. The Ace index was significantly lower in the PA_HFD group than in the HFD group (*p* < 0.01), and the Chao index showed a decreasing trend (*p* > 0.05) (Figure 6A,B). The Simpson and Shannon indices characterize the richness of microbial communities. The Shannon index of the PA_HFD group was significantly lower than that of the HFD group (*p* < 0.001), whereas the Simpson index was higher than that of the HFD group (*p* > 0.05) (Figure 6C,D). A total of 276 shared species and 684 endemic species were detected at the operational taxonomic unit level in all three groups (Figure 6E). The number of shared species was 70 in the PA group and 147 in the CON and HFD groups, whereas the number of shared species was 48 in the CON and HFD groups (Figure 6E). The microbial β-diversity did not significantly differ (Figure 6F).

### 3.7. Effect of PAs on the Microbial Composition of Mouse Colon

**Figure 7:**
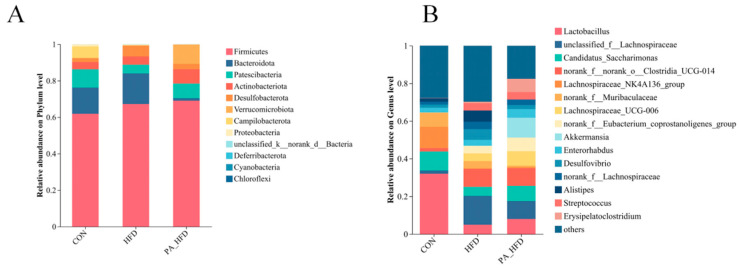
Effect of PAs on the microbial composition of mouse colon. (**A**) Phylum-level composition; (**B**) Genus-level composition. *n* = 6.

Analysis of the colonic microflora showed that the dominant phyla were *Firmicutes*, *Bacteroidota*, *Patescibacteria*, *Actinobacteriota*, and *Desulfobacterota*. Among them, *Firmicutes* and *Desulfobacterota* were significantly increased in the HFD group (*p* < 0.01), and *Actinobacteria* and *Desulfobacterota* were significantly increased in the PA_HFD group (*p* < 0.05) compared to those in the CON group. *Patescibacteria*, *Campilobacterota*, and *Proteobacteria* were significantly reduced in the HFD group (*p* < 0.01). However, the abundance of *Bacteroidota*, *Campilobacterota*, and *Proteobacteria* was significantly reduced in the PA_HFD group (*p* < 0.01). *Actinobacteriota* significantly increased (*p* < 0.05), and *Patescibacteria* was highly significantly increased (*p* < 0.01). *Bacteroidota*, *Desulfobacterota*, and *Proteobacteria* were significantly decreased in the PA_HFD group compared with those in the HFD group (*p* < 0.01) (Figure 7A). At the genus level, compared to the HFD group, the PA_HFD group exhibited a significantly higher abundance of *Candidatus_Saccharimonas*, *Lactobacillus*, *Akkermansia*, *Lachnospiraceae_UCG-006* (*p* < 0.01), *Lachnospiraceae_NK4A136_group* (*p* > 0.05), whereas *unclassified_f__Lachnospiraceae*, *Desulfovibrio*, *Alistipes*, *Bacteroides*, and *norank_f__Muribaculaceae* abundance was highly significantly decreased (*p* < 0.01), and *norank__f__Lachnospiraceae* abundance was significantly decreased (*p* < 0.05) (Figure 7B).

### 3.8. Effects of PAs on Metabolites in Mouse Colon

**Figure 8:**
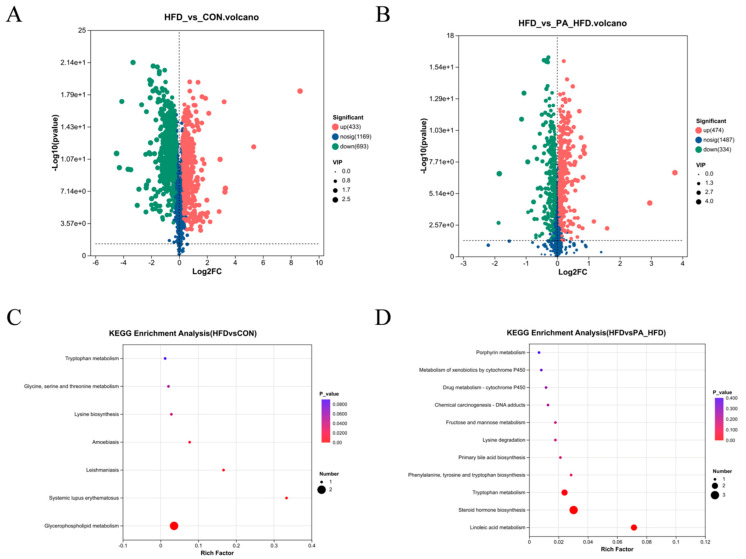
Effects of PAs on metabolites in the mouse colon. (**A**) Volcano plots of metabolites differing between CON and HFD groups; (**B**) Volcano plots of metabolites differing between HFD and PA_HFD groups; (**C**) KEGG pathway-enriched bubble plots between CON and HFD groups; (**D**) KEGG pathway-enriched bubble plots between HFD and PA_HFD groups. *n* = 6.

**Figure 9:**
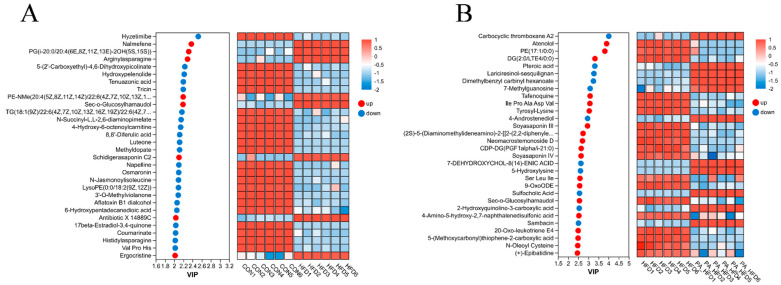
Effect of PAs on the levels of metabolites in the mouse colon. (**A**) Changes in metabolites between the CON and HFD groups; (**B**) Changes in metabolites between the HFD and PA_HFD groups. The figure shows the VIP plots between the CON group and the HFD group, and between the HFD group and the PA_HFD group. *n* = 6.

Metabolites with VIP > 1 and *p* < 0.05 were categorized as differential metabolites, and 8892 significantly differential metabolites were detected in all three groups. Metabolites were differentially upregulated and downregulated between the HFD group and the other two groups (Figure 8A,B). The trends in the expression of significantly differential metabolites are shown in Figure 9. Compared with the other two groups, the most abundant compounds in the HFD group were lipids and lipid-like molecules. In the CON and HFD groups, the differential metabolites were mainly concentrated in fatty acids and glycerophospholipids, where the HFD significantly downregulated osmaronin and 6-hydroxypentadecanedioic acid metabolites in fatty acids compared to the CON group. KEGG pathway enrichment analysis indicated that the glycerophospholipid metabolic pathway was enriched in the CON and HFD groups (Figure 8C), whereas linoleic acid metabolism, steroid hormone biosynthesis, and tryptophan metabolism were enriched in the HFD and PA_HFD groups (Figure 8D).

### 3.9. Correlation Analysis Between Differential Microflora and Metabolites in Colon and Inflammatory Factors in Hippocampal Tissue

**Figure 10:**
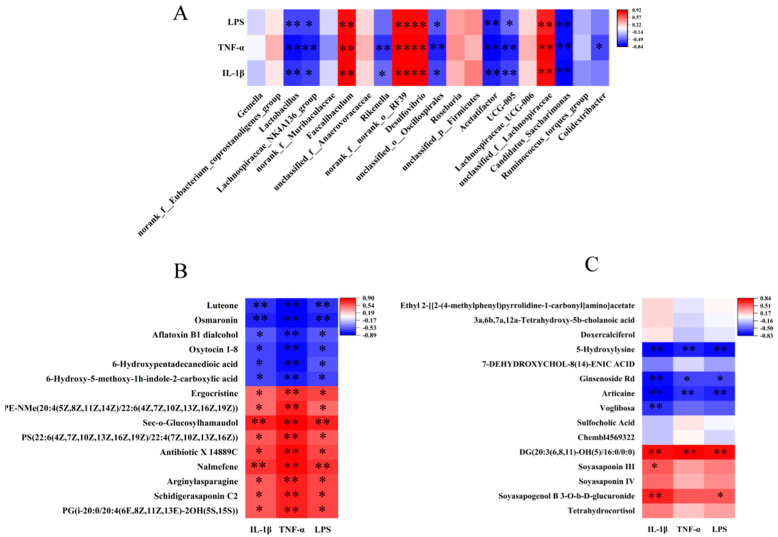
Correlation analyses among the microflora and metabolites in the colon and inflammatory factors in the hippocampal tissue. (**A**) Microflora and inflammatory factors; (**B**) CON and HFD groups; (**C**) HFD and PA_HFD groups. * *p* < 0.05, ** *p* < 0.01.

Spearman correlation analyses between genus-level differential intestinal flora and inflammatory factors showed that *Lactobacillus*, *Lachnospiraceae_NK4A136_group*, and *Candidatus_Saccharimonas* were negatively correlated with IL-1β, TNF-α, and LPS, whereas *Desulfovibrio* and *unclassified_f__Lachnospiraceae* were positively correlated with IL-1β, TNF-α, and LPS (Figure 10A). Correlation analysis also indicated that 15 metabolites were negatively correlated with IL-1β, TNF-α, and LPS in CON and HFD groups, such as luteone and oxytocin 1-8 (Figure 10B). In the HFD and PA_HFD groups, 5-hydroxylysine, ginsenoside Rd, and articaine were negatively correlated with IL-1β, TNF-α, and LPS, but DG was positively correlated with IL-1β, TNF-α, and LPS (Figure 10B). In addition, soyasapogenol B 3-O-b-D-glucuronide was positively correlated with IL-1β and LPS, and voglibosa was negatively correlated with IL-1β (Figure 10C).

## 4. Discussion

Obesity is a chronic metabolic disease caused by the excessive accumulation of fat and abnormal fat [33]. As dietary flavonoids, PAs have shown some preventive effects against obesity owing to their ability to prevent increases in body weight and adipose tissue mass [34]. PAs significantly ameliorated HFD-induced obesity and obesity-associated symptoms in mice, including reduced body weight gain, improved lipid profiles, and increased energy expenditure [28]. Grape seed-derived PAs (0.5 g/kg) inhibit weight gain by reducing food intake and activating energy expenditure in subcutaneous adipose tissue [35]. In the present study, PAs significantly reduced the body weight of HFD-fed mice. Furthermore, although cognitive function in mice showed some improvement in this study, the effect was not statistically significant. This may be attributed to the relatively short duration of PA treatment. A study investigating the polyphenol catechin demonstrated that long-term treatment over 5 months significantly improved cognitive function in mice [36].

When cholesterol levels in the body exceed the normal range, the organism develops hypercholesterolemia, a condition that often triggers metabolic and cardiovascular diseases [37]. A growing body of research has confirmed that the development of cognitive impairment may be associated with high plasma cholesterol levels and obesity [38]. PAs with different degrees of polymerization were effective at reducing TC, TG, and LDL levels in serum and liver tissues [39]. Consistent with the results of previous studies, PAs significantly reduced the serum TC and LDL levels in the mice in our study. LDL is a natural lipid-transporting protein in human plasma, and the promotion of excess LDL clearance is an effective clinical approach for the treatment of hyperlipidemia [40]. In addition, PAs increased serum HDL levels in mice. HDL represents a group of heterogeneous particles circulating in the blood and is a key component of glucose metabolism [41]. Grape seed PAs can reduce the serum levels of TC, TG, and LDL cholesterol and increase the serum levels of HDL cholesterol [42]. High plasma TG levels and chronic inflammation are associated with metabolic diseases, and TG and its major circulating transporter lipoproteins (VLDL) appear to be associated with inflammation [43]. In the present study, the TG content was higher in both the HFD and PA_HFD groups than in the CON group, which may be related to the inflammation observed in HFD mice.

Morphological analysis of the abdominal fat revealed that PAs alleviated HFD-induced breakage, fusion, and inflammatory cell infiltration of abdominal fat vacuoles in mice. Overweight and obesity increase the risk of liver disease, and 65% of elevated ALT levels are caused by overweight and obesity [44]. Serum ALT, a widely used clinical indicator of liver disease, is a marker of liver integrity or hepatocellular injury and increases the risk of metabolic disorders [45]. Elevated serum AST levels have been used to detect liver, heart, brain, and kidney disease [46]. In the present study, PAs reduced serum ALT and AST levels in HFD mice. In addition, histomorphometric analysis of the liver revealed that the livers of mice in the HFD group showed increased adipocytosis, steatosis, and hepatocellular edema, which were alleviated by PAs, suggesting that PAs may attenuate HFD-induced liver injury. This suggests that PAs alleviate HFD-induced cognitive deficits in mice by attenuating obesity, dyslipidemia, and inflammation.

Cognitive impairment and neuroinflammation are complications of metabolic diseases induced by HFDs and high-cholesterol diets [47]. In the central nervous system, mild cognitive impairment can be attributed to obesity-induced changes in hippocampal structure and function [48]. A short-term HFD is associated with inflammation in the hippocampus of young rats [49]. In addition, inflammatory cytokines, such as IL-6 and TNF-α, secreted by adipocytes and macrophages in adipose tissue have been shown to activate inflammatory responses [50]. Dietary anthocyanins attenuated hippocampal inflammation in mice induced by HFD [51]. A blueberry supplement containing anthocyanins reduced the expression of *TNF-α* and *IL-1β* genes in HFD-fed male rats [52]. Procyanidin A1 also attenuated the inflammatory response induced by LPS via NF-κB, MAPK and Nrf2/HO-1 pathways in RAW264.7 cells [53]. Similarly to the results of previous studies, PAs significantly reduced IL-1β, TNF-α, and LPS content in mice in the present experiment, suggesting that PAs may alleviate the cognitive deficits induced by HFD in mice by attenuating inflammation in hippocampal tissues.

Autophagy is a complex degradation pathway responsible for the removal of damaged and dysfunctional organelles [54]. HFD-induced obesity activates autophagy in adipocytes in a tissue-specific manner [55]. Obesity-induced chronic low-grade inflammation can be modulated by the pro- and anti-inflammatory effects of autophagic mechanisms [56]. HFD may inhibit the expression of the SIRT1/AMPK pathway and disrupt the autophagic pathway, leading to tau hyperphosphorylation and synaptic dysfunction, ultimately leading to cognitive decline [57]. FGF21 attenuates HFD-induced cognitive dysfunction in obese mice through metabolic regulation and anti-inflammation [58]. In the present experiment, PAs significantly increased Sirt1 and FGF21 expression in hippocampal tissues. Among autophagy-related genes, those encoding LC3 and Beclin1-related proteins are involved in the initial stages of autophagy [59]. In a previous study, a short-term HFD was shown to induce significant upregulation of LC3 mRNA expression in the cartilage of male rats [60]. However, in another study, the HFD reduced the expression of LC3B-II, a marker of autophagosome formation [61]. The differences in the expression of the autophagy marker LC3 may stem from variations in the tissues examined. Studies have found that suppressing autophagy expression can inhibit apoptosis in hippocampal neurons of mice [62].

In the present study, PAs downregulated Lc3, Beclin, and ULK1 expression in HFD mice while upregulating Atg3 and p62 expression. Gandulin alleviates oxidative stress and autophagy to reduce cognitive dysfunction in a mouse model of Wilson’s disease by modulating the p62/Nrf2 signaling pathway [63]. ATG3 is associated with the development of steatosis [64], whereas ULK1 is associated with lipid accumulation in the liver [65]. This suggests that PAs may mitigate inflammation and cognitive impairment induced by HFD in mice by regulating autophagy-related genes.

An HFD alters the gut microflora and promotes inflammation and cognitive impairment associated with obesity [66]. Gut flora have been linked to a variety of central nervous system disorders, and gut dysbiosis, cognitive dysfunction, and inflammatory responses are associated with each other [27]. The gut microbiota plays an important role in the onset and progression of inflammatory and psychiatric disorders through its interactions with the brain and neuroinflammation [67]. More importantly, gut microbiota, microbial metabolites, and cognitive decline are strongly associated [68]. The gut flora plays an unexpected key role in HFD-induced cognitive dysfunction [69]. *Actinobacteria* abundance is positively correlated with learning and memory abilities, with an increase in *Actinobacteria* abundance improving learning and memory disorders [70]. In the present study, the PAs increased the abundance of *Actinobacteria*. *Candidatus_Saccharimonas*, *Lactobacillus*, and *Lachnospiraceae_UCG-006* are beneficial microflora [71,72,73] associated with inflammation, lipid metabolism, and oxidative stress [74,75,76]. *Candidatus_Saccharimonas* and *Lactobacillus* are involved in regulating the gut microbiota induced by an HFD [77,78]. *Candidatus_Saccharimonas* and *Lachnospiraceae_UCG-006* may affect cognitive impairment in Alzheimer’s disease mice with metabolic disorders [79]. The relative abundance of intestinal *Lachnospiraceae_UCG-006* and other genera was reduced in mice fed an HFD [80]. Obese mice fed an HFD exhibited significant cognitive deficits and inflammation, as well as significant changes in the gut microflora, such as *Akkermansia* [81]. While improving the cognitive behavioral deficits induced by HFD in mice, gut microflora, such as *Akkermansia* in mice, showed an upregulation trend [82].

Similarly to the results of previous studies, in our experiment, PAs significantly increased the abundance of *Candidatus_Saccharimonas*, *Lactobacillus*, *Lachnospiraceae_UCG-006*, and *Akkermansia* in the colons of mice fed an HFD. Correlation analysis with hippocampal tissue inflammatory factors showed that *Lactobacillus*, *Lachnospiraceae_NK4A136_group*, and *Candidatus_Saccharimonas* were negatively correlated with IL-1β, TNF-α, and LPS. In addition, HFD influenced *Lachnospiraceae_NK4A136_group*, *unclassified__f__Lachnospiraceae*, *Lactobacillus*, and *norank__f__Lachnospiraceae*, all of which have some influential role in relieving nonalcoholic fatty liver disease [83]. PAs significantly reduced *unclassified__f__Lachnospiraceae* and *norank__f__Lachnospiraceae* abundance. Upregulated *Akkermansia* and downregulated *Desulfovibrio* may be associated with cognitive impairment and inflammation in mice [84]. The metabolite soyasapogenol B 3-O-β-D-glucuronide, which is positively correlated with *Desulfovibrio* and inflammatory factors, may cross the blood–brain barrier and contribute to cognitive dysfunction in HFD-fed mice. Studies have shown that luteolin-7-O-β-d-glucuronide can cross the blood–brain barrier and alleviate cerebral ischemia–reperfusion injury in rats [85]. The differences in the effects of these metabolites arise from variations in their molecular structures, which alter their biological properties. HFD may increase the abundance of the pro-inflammatory bacterium *Desulfovibrio* in the colon of mice [86]. *Alistipes* is also associated with inflammation, obesity, and depression [87]. *Alistipes* is positively correlated with IL-6 [88], and chronic psychological stress leads to cognitive impairment while increasing the relative abundance of genera such as *Alistipes* [89]. Similarly to the results of previous studies, PAs significantly reduced *Desulfovibrio*, *Alistipes*, and *Bacteroides* abundance in our experiment, and *Desulfovibrio* and *unclassified_f__Lachnospiraceae* were positively correlated with mouse hippocampal tissue IL-1β, TNF-α, and LPS. These results suggest that PAs attenuate HFD-induced inflammation and cognitive deficits in mice by increasing the abundance of beneficial colonic microflora.

Gut metabolites are produced by both the host and gut microbiota and play a crucial role in maintaining host health. To identify the differential metabolites involved in the beneficial effects of PAs, we analyzed the changes in metabolite profiles and metabolic pathways using colonic contents. Fatty acids play an important role in the regulation of glucose metabolism and inflammation [90]. HFD can alter fatty acids in mice [91], which are associated with food intake, body weight gain rate, and lipid homeostasis [92]. In the present study, HFD significantly downregulated osmaronin and 6-hydroxypentadecanedioic acid metabolites (which show antimicrobial activity) [93]. HFD significantly upregulated PE-NMe (20:4/22:6) and PS (22:6/22:4) metabolites. Disorders of glycerophospholipid and fatty acid metabolism have been found to be directly related to the occurrence and development of hyperlipidemia. HFD regulates glycerophospholipid synthesis and lipid metabolism related to the occurrence and development of hyperlipidemia [94,95]. In the present study, the HFD significantly upregulated the metabolites tetrahydrocortisol and doxercalciferol and significantly downregulated doxercalciferol (steroid and steroid derivative compounds) compared with the PA_HFD group. Thus, the effects of PAs on HFD-induced obesity in mice may be related to changes in the levels of fatty acids, glycerophospholipids, steroids, and steroid derivatives.

HFD-induced colonic metabolites in mice were enriched in the glycerophospholipid metabolic pathway. In contrast, colonic metabolites from PA-treated mice were enriched in the steroid hormone biosynthesis metabolic pathway. Glycerophospholipids, as lipid molecules, can be involved in lipid synthesis and degradation through metabolic pathways. Simultaneously, any change in glycerophospholipid homeostasis adversely affects brain function [96], and dysregulated glycerophospholipid metabolism reflects systemic changes induced by inflammatory responses and oxidative stress [97]. In the biosynthetic metabolic pathway of steroid hormones, PAs significantly upregulate the levels of 2-methoxyestradiol, which inhibits adipose tissue macrophage infiltration and immune phenotypes by modulating HFD-induced obesity [98]. Thus, HFD may cause disorders of colonic metabolites in mice through the glycerophospholipid metabolism pathway, and PAs may regulate steroid hormone biosynthesis by modulating 2-methoxyestradiol levels, which alleviate cognitive impairment in mice. The 2-methoxyestradiol regulates obesity and lipid metabolism induced by HFD in mice, which alleviates cognitive impairment.

## 5. Conclusions

PAs can ameliorate neuroinflammation and dyslipidemia by regulating the abundance of colonic microflora and by improving glycerol-phospholipid metabolism and steroid hormone biosynthesis in HFD mice. However, its improvement on the cognitive function of mice was not statistically significant. This lays the foundation for future exploration of more polyphenols to mitigate neuroinflammation induced by obesity and partially improve cognitive function.

## Figures and Tables

**Table 1:** Primer sequences.

Genes	Accession No.	Primer Sequences (5′-3′)	Size/bp
*ULK1*	XM_011249420.4	F: TGGAGGTGGCCGTCAAATG R: CGCATAGTGTGCAGGTAGTC	202
*Sirt1*	NM_001159589.2	F: TGATTGGCACCGATCCTCG R: CCACAGCGTCATATCATCCAG	86
*Becline-1*	NM_001359821.1	F: ATGGAGGGGTCTAAGGCGTC R: TGGGCTGTGGTAAGTAATGGA	149
*P62*	XM_036156414.1	F: GAGGCACCCCGAAACATGG R: ACTTATAGCGAGTTCCCACCA	79
*Atg3*	NM_026402.3	F: CTGGAGATCACTTAGTCCACCA R: GTCGGAAGATATGCCTTCACTTT	82
*Lc3*	NM_026160.5	F: GCTCGCTGCTGTCTAGATGT R: GAAACAGCTCTCCAGTCGCT	102
*FGF21*	NM_020013.4	F: CACCGGAGTCAGAACACAATTC R: AGGGATGGGGTATGCTTGGTA	184
*ACTB*	NM_007393.5	F: ATATCGCTGCGCTGGTCG R: GATCTTCTCCATGTCGTCCC	245

## Data Availability

Raw data were uploaded to the NCBI BioProject database (https://www.ncbi.nlm.nih.gov/bioproject/, accession number: PRJNA1296861) and analyzed using the Meggie BioCloud platform (https://cloud.majorbio.com).

## Abbreviations

The following abbreviations are used in this manuscript:

ALT	alanine aminotransferase
AST	aspartate aminotransferase
BG	blood glucose
CON	control
H&E	hematoxylin and eosin
HDL	high-density lipoprotein
HFD	high-fat diet
IL-1β	interleukin-1β
LDL	low-density lipoprotein
LPS	lipopolysaccharide
PA	proanthocyanidin
TC	total cholesterol
TG	triglyceride
TNF-α	tumor necrosis factor
